# Advances in Regenerative and Reconstructive Medicine in the Prevention and Treatment of Bone Infections

**DOI:** 10.3390/biology13080605

**Published:** 2024-08-10

**Authors:** Leticia Ramos Dantas, Gabriel Burato Ortis, Paula Hansen Suss, Felipe Francisco Tuon

**Affiliations:** Laboratory of Emerging Infectious Diseases, School of Medicine, Pontifícia Universidade Católica do Paraná, Curitiba 80215-901, Paraná, Brazil; leticia.dantas@pucpr.edu.br (L.R.D.); gabriel.ortis@pucpr.edu.br (G.B.O.); p.hansen@pucpr.br (P.H.S.)

**Keywords:** biofilm, silver, bone, nanotechnology, impregnation, bacteria

## Abstract

**Simple Summary:**

Regenerative medicine specifically aims to harness the body’s innate healing abilities through therapies that utilize cells and scaffolds derived from these materials to facilitate tissue repair and regeneration. For instance, in bone repair, addressing defects often involves the use of bone grafts, which are indispensable in medical and dental practices globally. Bovine bone scaffolds are frequently preferred over autografts due to their ability to mitigate surgical risks and reduce costs. Enhancing these bone substitutes with antimicrobial properties, particularly through metals like zinc, copper, and silver, holds promise in preventing infections during surgical procedures. Silver nanoparticles, known for their broad-spectrum antimicrobial activity, and zinc nanoparticles, which aid in infection prevention while supporting bone healing, exemplify these advancements in medical technology.

**Abstract:**

Reconstructive and regenerative medicine are critical disciplines dedicated to restoring tissues and organs affected by injury, disease, or congenital anomalies. These fields rely on biomaterials like synthetic polymers, metals, ceramics, and biological tissues to create substitutes that integrate seamlessly with the body. Personalized implants and prosthetics, designed using advanced imaging and computer-assisted techniques, ensure optimal functionality and fit. Regenerative medicine focuses on stimulating natural healing mechanisms through cellular therapies and biomaterial scaffolds, enhancing tissue regeneration. In bone repair, addressing defects requires advanced solutions such as bone grafts, essential in medical and dental practices worldwide. Bovine bone scaffolds offer advantages over autogenous grafts, reducing surgical risks and costs. Incorporating antimicrobial properties into bone substitutes, particularly with metals like zinc, copper, and silver, shows promise in preventing infections associated with graft procedures. Silver nanoparticles exhibit robust antimicrobial efficacy, while zinc nanoparticles aid in infection prevention and support bone healing; 3D printing technology facilitates the production of customized implants and scaffolds, revolutionizing treatment approaches across medical disciplines. In this review, we discuss the primary biomaterials and their association with antimicrobial agents.

## 1. Introduction

Reconstructive and regenerative medicine represent pivotal disciplines within medical science dedicated to restoring the form and function of compromised tissues and organs. The fundamental objective is to rehabilitate tissues and organs impaired by injury, disease, or congenital anomalies. This necessitates the use of tissue substitutes composed of biomaterials [1].

Biomaterials encompass a diverse range including synthetic polymers, metals, ceramics, and biological tissues or cells, crucial for reconstructing or replacing damaged tissues [2]. Integration with the body’s tissues is paramount to preventing adverse immune responses or rejection. Advanced methodologies such as precise imaging (e.g., CT scans and MRI) and computer-assisted design enable the creation of personalized implants or prosthetics tailored to individual patients [3]. Durability is essential to withstand biological conditions and mechanical stresses over prolonged periods.

By contrast, regenerative medicine focuses on stimulating intrinsic repair mechanisms rather than solely substituting damaged tissues [4]. This approach aims to harness the body’s innate capacity for healing. Cellular therapies, utilizing stem cells, progenitor cells, or other cellular components, are pivotal in promoting tissue regeneration and repair [5]. Concurrently, biomaterial scaffolds play a crucial role in supporting cellular growth and facilitating the organization of cells into functional tissues [6]. The synergistic combination of scaffolds and cellular therapies exemplifies the potential for overlapping therapeutic modalities [7].

Bioactive molecules, including growth factors, cytokines, and other signaling molecules, play integral roles in promoting cellular proliferation, differentiation, and ultimately tissue regeneration [8]. These fields are advancing rapidly through interdisciplinary collaboration among scientists, engineers, clinicians, and biotechnologists, offering promising avenues for patients confronting complex tissue damage or organ dysfunction.

## 2. Bone Repair

Repairing and reconstructing bone defects is a critical necessity in both medical and dental practices. Bone grafts are essential for addressing volumetric bone loss resulting from various conditions such as periodontal disease, trauma, tooth extraction requiring alveolar ridge preservation, maxillary sinus augmentation, ridge regeneration to enhance bone height and thickness, extensive reconstructions of atrophic maxillae and mandibles, cleft palate treatments, and bone defects in areas including oral, maxillofacial, cranial base, spinal fusion, and orthopedic (congenital malformations) regions [9,10,11]. Bone tissue transplantation ranks as the second most transplanted tissue globally, with countries such as the United States and several European nations facing the highest demand. Annually, these regions collectively require approximately half a million bone grafting procedures to address various medical needs [12]. In the United States, allografts are the predominant type of bone graft used, whereas in Europe, bovine xenografts are more commonly employed for bone grafting procedures [13].

Bovine bone scaffolds provide an alternative to autogenous grafts, which necessitate harvesting bone from a second surgical site. The need for an additional surgical procedure to obtain bone grafts increases morbidity, the risk of infection, hemorrhage, and the potential for peripheral nerve lesions [14]. Bovine bone grafts offer several advantages: they have lower production costs compared with other types of grafts, are readily available on the market, and exhibit favorable characteristics, such as good osteoinduction, osteoconduction, and mechanical stability [13].

## 3. Bone Repair and Antimicrobial Activity

The development of bone substitutes with antimicrobial properties is crucial in both orthopedics and dentistry. In orthopedics, these substitutes are utilized to fill spaces prone to biofilm formation, addressing infection risks. In dentistry, particularly in implantology, they mitigate the potential contamination risks associated with dental implants [15].

Several molecules have been tested for this purpose, including metals with antimicrobial activity, whether in their salt form or as nanoparticles, as well as classical antimicrobials that have been extensively studied in intravenous or oral formulations [16,17,18,19,20].

**Figure 1:**
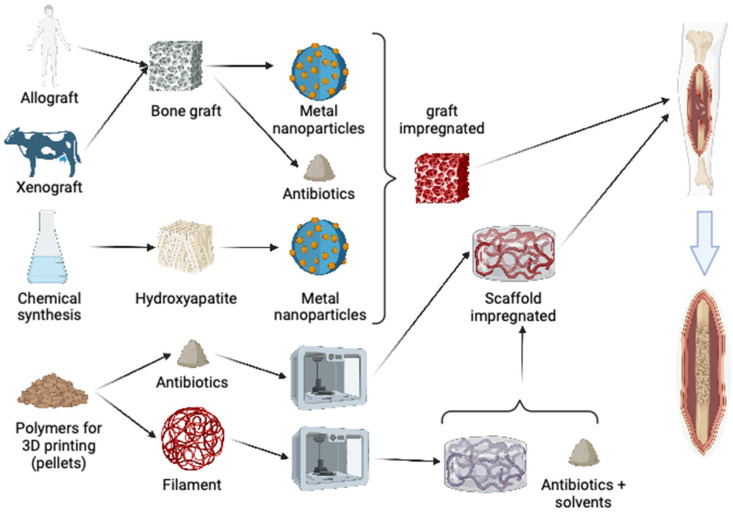
A diagram demonstrating multiple options for doping bone grafts or polymers for 3D printing using metal nanoparticles or antibiotics in bone reconstruction.

In theory, impregnating bone scaffolds can reduce both intraoperative and postoperative infection rates, thereby lowering the risks associated with bone volume loss and chronic infectious processes like osteomyelitis [21]. However, the impregnation of bone grafts with nanometals has been inadequately investigated, with a lack of microbiological studies focusing on biofilms, including those involving multidrug-resistant bacteria. The existing literature on bone grafts and nanometals primarily emphasizes the development of implants and grafts using hydroxyapatites and other synthetic materials [22] (Figure 1).

## 4. Metals and Nanoparticles

Among metals, zinc, copper, and silver are the most commonly studied for bone repair. Silver was chosen as a potential antimicrobial agent due to its broad spectrum of activity against both Gram-negative and Gram-positive bacteria. It can be transformed into nanoparticles to enhance its effectiveness [23]. There is also theoretical potential for improved osteointegration. Importantly, silver does not share chemical similarities with antibiotics used in treatments, thereby reducing the risk of inducing resistance or selecting multidrug-resistant bacteria through modification of the microbiota. [24].

**Figure 2:**
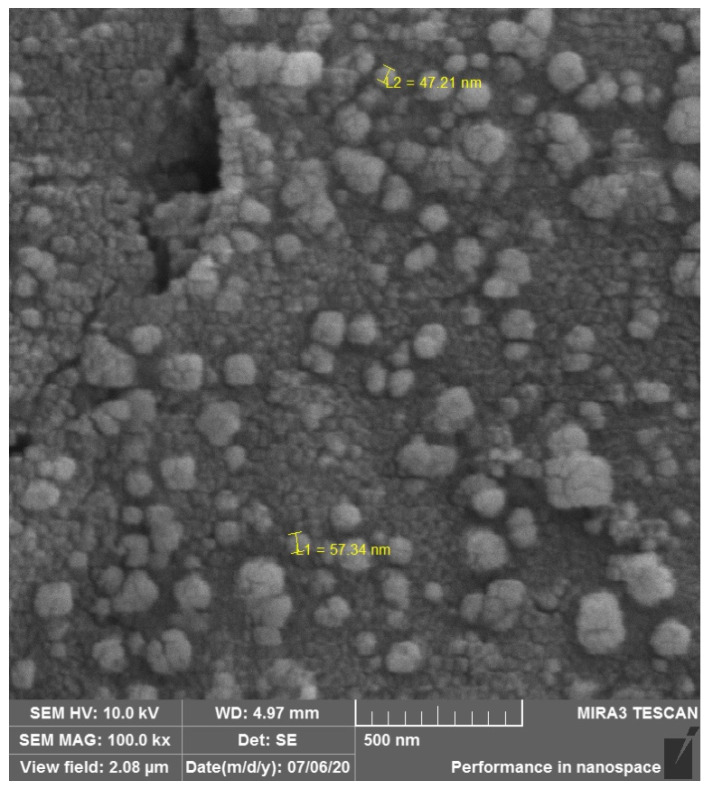
Silver nanoparticles on bone surface used for orthopedic graft.

In a previous study, we tested several pathogens, including multidrug-resistant microorganisms, and found them to be highly susceptible to silver. Whether using silver nitrate or silver nanoparticles (AgNPs), the antimicrobial activity remained sustained [25]. Due to potential variations in nanoparticle preparation during production, it is challenging to perform comparative analyses among existing publications. The synthesis process varies, silver concentration fluctuates, nanoparticle sizes differ, and standardizing synthesis is difficult due to numerous extrinsic factors that can disrupt the process (Figure 2). Moreover, methods of tissue impregnation vary, further complicating envisioning this as a viable therapy in the near term. Mathur et al. described the antibacterial action of AgNPs by penetrating the microorganism’s cell membrane, increasing its permeability, ultimately causing membrane rupture and cell death [26]. Another mechanism described involves the formation of free radicals that inflict damage on the cell membrane. This process leads to increased membrane porosity and eventual cell lysis [27]. Toxicity is not limited to bacterial cells but also extends to eukaryotic cells, as nanoparticles can exhibit varying degrees of cytotoxicity [28,29]. The cytotoxicity assay revealed moderate toxicity in the AgNP-impregnated bone scaffold we developed, consistent with findings from previous studies cited. Another concern associated with the use of silver is its potential for discoloration, which may stain teeth or skin if directly applied to these tissues [30]. The biocompatibility of silver-containing nanoparticles has already been demonstrated, reinforcing the potential for their use in medical practice [31].

In recent years, there has been a notable rise in studies investigating the antibiofilm activity of AgNPs. These studies have explored the efficacy of AgNPs in combination with metallic surfaces, such as titanium compounds, to prevent infections associated with orthopedic prostheses and dental implants [32]. In the realm of dentistry, incorporating AgNPs into dental restorations has demonstrated efficacy against *Streptococcus* biofilms, the predominant pathogens linked to dental plaque and subsequent caries development [33]. Another strategy to combat biofilms involves combining AgNPs with biocompatible materials such as chitosan. This approach allows AgNPs to be integrated into hydrogels or incorporated into 3D printing processes using bioprinters. These advancements facilitate the development of therapeutic or preventive wound dressings with enhanced antimicrobial properties [34,35].

Smaller particles have a larger surface area, which facilitates the release of a greater amount of silver ions. These ions are responsible for the bactericidal action, which is considered the most effective mechanism for combating bacteria [36]. In this context, smaller particles are anticipated to exhibit a stronger antimicrobial effect compared to larger particles and particle aggregates. This attribute could prove highly advantageous if our AgNP-treated graft is employed as an adjunct in chronic osteomyelitis cases that necessitate filling [37]. Silver nanoparticles, despite their cytotoxic potential, have been shown to stimulate the development of osteoblasts and can be linked with enhanced osteogenesis [38].

Zinc has been studied for its potential in preventing infections in bone grafts [39]. Zinc possesses well-known antimicrobial properties that can help reduce the risk of infection at bone graft sites [40]. Studies have demonstrated that zinc plays a crucial role in collagen synthesis and wound healing, which can be beneficial for promoting graft integration and postoperative recovery [41]. Materials containing zinc have been reported to enhance bone repair by promoting cell proliferation, stimulating osteogenic activity, fostering angiogenesis, and inhibiting osteoclast differentiation. These properties contribute significantly to the overall process of bone healing and regeneration [40].

The mechanisms through which zinc may act to prevent infections include its ability to modulate local immune responses, enhance the activity of defense cells such as macrophages, and improve the integrity of the epithelial barrier [42]. Several clinical and experimental studies have investigated the use of zinc for infection prevention in surgical procedures, including bone grafts [43]. These studies typically assess the effectiveness of zinc in reducing infection rates and improving postoperative outcomes [44].

In the same vein as described for AgNPs, nanoparticles containing zinc (ZnNPs) offer advantages over isolated zinc metal in impregnation processes. Biomimetic nanofibrous scaffolds made of Poly ε-caprolactone with ZnNP were studied to determine the optimal concentration range. The research demonstrated biocompatibility and supported osteoregeneration, highlighting the potential benefits of incorporating zinc oxide nanoparticles into scaffold materials for enhanced biomedical applications [45].

ZnNPs also exhibit antibiofilm activity against medically important bacteria such as *Staphylococcus aureus*, *Klebsiella oxytoca*, and *Pseudomonas aeruginosa*, prevalent in developed countries. While not yet studied in bone grafts, previous research has shown promising results for various applications [46].

Copper has also been investigated for its potential in preventing infections in bone grafts [47]. Copper possesses natural antimicrobial properties that are effective against a broad spectrum of microorganisms, including bacteria, viruses, and fungi [48]. Biomaterials incorporating copper, such as copper-coated implants and composite materials that release copper gradually, have been studied for their ability to prevent infections. In addition to its antimicrobial properties, copper can promote angiogenesis and osteogenesis, additional crucial properties in tissue regeneration [49]. In their study, Ryan et al. evaluated the effects of copper in an osteomyelitis model, an analysis not commonly included in most in vivo studies [49].

Copper nanoparticles (CuNPs) have been studied to enhance their antimicrobial activity while reducing toxicity through sustained release [50]. Some studies in humans and robust in vivo models involving scaffolds have been published, including periosteal tissues such as tendons [51,52,53,54,55].

## 5. Antibiotics

The combination of antibiotics with scaffolds presents some challenges due to the properties of both bone and antibiotics, making their physical or chemical adsorption difficult and possibly requiring an anchoring bridge with another molecule. A study a using graft combined with gentamicin and calcium sulfate, despite exhibiting good antimicrobial activity, showed inert capacity on the graft [56]. Conversely, an in vivo model utilizing a vancomycin release system focused more on infection prevention management, demonstrating not only effective antimicrobial activity but also a role that did not interfere with osteointegration [57]. In these studies, unlike metals, the effect of antibiotics on not hindering osteointegration should be evaluated, as their hydrophilic or lipophilic characteristics may attract fluids or lipids that interfere with graft osteointegration [56,58].

Oxacillin, an antibiotic with anti-*Staphylococcus* activity, was combined with hydroxyapatite and demonstrated antimicrobial activity, serving as dead-space filler, which is common in orthopedic infections requiring debridement to remove necrosis [59].

## 6. Polymers for 3D Printing

A range of publications and new products have been developed as antimicrobial release systems, but scaffolds with antibiotics lack more advanced studies [58]. Ceramic scaffolds for dead-space filling have also been described. In a systematic review, ceramics doped with vancomycin, gentamicin, and quinolones were evaluated, all showing antimicrobial activity, with some achieving complete and others partial healing in different animal models [60,61,62].

Three-dimensional printing is a technology that enables the creation of three-dimensional objects from a digital model. It operates on an additive manufacturing model, meaning it builds objects layer by layer without the need for molds, thereby reducing material waste [63]. Compared with traditional subtractive manufacturing, additive manufacturing offers a cheaper and faster technique for producing complex shapes. This method of production allows for high customization possibilities [64]. Furthermore, 3D printing enables on-demand production of spare parts and customized components, minimizing the need for large inventory stocks. This technology has found applications across a wide range of industries including medicine, architecture, automotive, aerospace, and many others. In medicine, for instance, 3D printing is used to create personalized prosthetics, medical implants, and anatomical models for surgical planning [65,66,67]. It is important to clarify that our focus is on the printing of thermoplastic polymers, although many printing methods exist, their application is still limited due to the lack of standardization [68].

The polymers used in 3D printing vary widely, but few possess the necessary biocompatibility for medical use. Among them are Polylactic Acid (PLA), Polyethylene Terephthalate Glycol-modified (PET-G), Thermoplastic Polyurethane (TPU), Polycaprolactone (PCL), and others that are less studied [69,70,71]. The advancement of 3D-printed bone reconstruction has been substantial in the past decade, yet practical implementation faces regulatory barriers in healthcare due to residues often present in their production, necessitating medical-grade printing filaments, which are currently limited in availability.

Polymers, generally hydrophilic, also pose challenges for doping with antimicrobials, requiring treatment with solvents that may be toxic to health [72,73]. A biomaterial that has attracted considerable interest for clinical applications due to its mechanobiological properties is polylactic acid (PLA), commonly used in additive manufacturing. PLA is a biodegradable and biocompatible thermoplastic derived from renewable sources like corn starch and sugarcane. [74]. Numerous studies have confirmed the biocompatibility of polylactic acid (PLA), demonstrating the absence of cytotoxic effects. [75]. For example, Chou et al. conducted experiments using custom-printed PLA cages to address segmental femoral bone defects in rabbits, which showed favorable tissue growth outcomes. The study concluded that the availability of cages with various geometric configurations could potentially benefit the treatment of significant segmental bone defects in humans [76].

**Figure 3:**
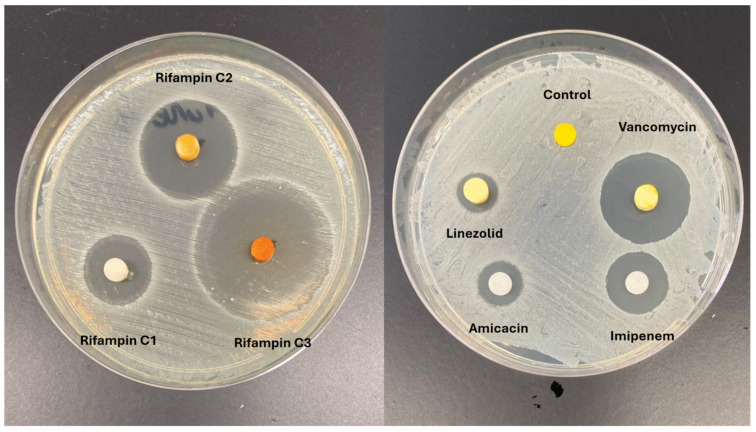
Antibiotic-impregnated PLA models with *Staphylococcus aureus* test.

**Figure 4:**
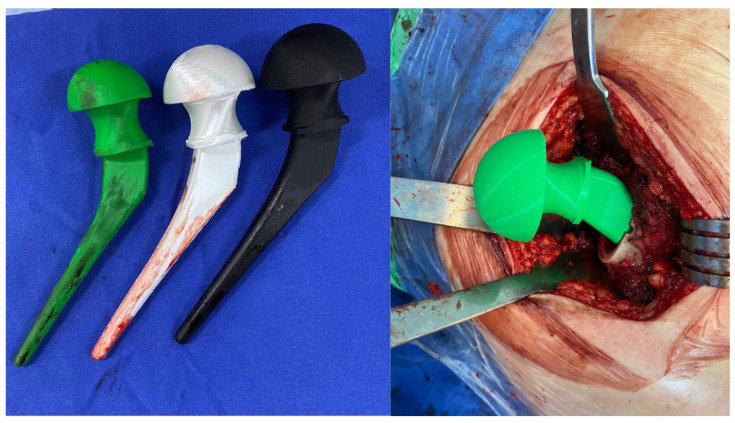
Implants with PLA impregnated with antibiotics tested during surgery for hip replacement.

Vancomycin and gentamycin stand out among antibiotics as a promising candidate for integration into thermoplastics due to its exceptional thermostability, long-term stability, and minimal association with tissue damage [77,78,79,80,81,82]. However, incorporating these into PLA presents a challenge because are hydrophilic, whereas PLA is hydrophobic [83]. In this scenario, surface modification of PLA becomes necessary to facilitate the impregnation of the antibiotic (Figure 3). One potential approach is to incorporate the drug into PLA through additive manufacturing processes involving high temperatures. The potential to incorporate antibiotics into PLA extends its utility in developing implants or temporary spacers for managing orthopedic infections [74,84]. Moreover, PLA employed in 3D printing technology facilitates the creation of personalized spacers, a critical component in addressing substantial bone defects (Figure 4) [85,86]. The use of pellets and using the extrusion combined with the antibiotic is an option to produce PLA filaments with vancomycin or gentamycin. This technique can also be used in new 3D printers to directly extrude the PLA pellets during printing.

In the case of TPU, another type of material that offers a certain degree of flexibility, its use is not recommended in bone grafts. However, it can be employed as an interface in joints considering its elasticity, such as a ligament, meniscus, or other structures that require flexibility. Its impregnation with antimicrobials has been evaluated for use in urinary catheters [87]. Another polymer widely used in medical devices is PET-G. However, we did not find studies on the combination of antibiotics with PET-G, opening up a range of opportunities for research and new models.

PCL has been extensively studied in combination with antibiotics, primarily vancomycin. Vancomycin has been combined with PCL in dicalcium phosphate or tricalcium phosphate beads for the treatment of osteomyelitis [88]. These beads can fill dead spaces, releasing antibiotics, and benefit from being absorbable, thus not requiring removal. When transformed into nanofibers in a mesh format, PCL can be placed in periprosthetic areas, aiding in the treatment of infections associated with orthopedic prostheses [89]. PCL combined with vancomycin has shown sustained antimicrobial activity, including anti-biofilm properties [90].

Another application of PCL involves its formulation into pellets combined with antibiotics and other elements, enabling 3D printing through direct extrusion, thereby facilitating its use in personalized reconstructive medicine [91]. PCL has also been tested as a scaffold for bone reconstruction [92]. In one study, PCL combined with vancomycin was also paired with osteogenesis-stimulating factors, enhancing interest in these combinations not only for treatment but also for bone remodeling [93].

PCL has also been combined with gentamicin, another thermally stable antibiotic suitable for 3D printing [94]. Furthermore, combining PCL with organic components such as collagen enhances the biocompatibility of the scaffold, noting that collagen constitutes 30% of bone composition [95].

## 7. Tissue Engineering In Situ

Recent clinical studies have demonstrated the effectiveness of intraoperative tissue engineering performed on-site [96]. Traditionally, “tissue engineering in situ” refers to various methods of developing grafts that will mature into functional tissue within the recipient’s body, utilizing its inherent regenerative capabilities [97]. However, the term “tissue engineering in situ” should specifically refer to the process of creating and implanting tissue-engineered grafts during the same surgical procedure. The key benefit of in situ tissue-engineered grafts is their immediate availability, unlike traditional tissue engineering, which requires significant time and resources for in vitro cell culture. Notably, grafts engineered during surgery can be enhanced with cells harvested intraoperatively with minimal handling [98]. The application of antimicrobial components in in situ tissue engineering is not yet well documented and requires further study to address issues related to graft loss due to handling and the risk of infection.

## 8. Challenges

Biomaterials impregnated with antibiotics face several challenges that can impact their effectiveness and safety. Over time, bacteria can develop resistance to antibiotics. If antibiotics are released from biomaterials, there is a risk of contributing to the development of resistant bacterial strains, which can complicate future treatments [99]. Achieving a controlled and sustained release of antibiotics from biomaterials is hard to define. The release rate must be carefully managed to ensure that therapeutic levels are maintained for an appropriate duration without causing toxicity or ineffective treatment. Our group has encountered difficulties in assessing the release of silver nanoparticles. On the other hand, this evaluation is less problematic for materials impregnated with antimicrobials, as it is easier to measure the release of these agents. However, the release rate of drugs in biomaterials is a complex issue, as it depends on the physicochemical interactions between the drugs and the material matrix. These interactions can vary depending on the type of biomaterial and the drug used, making the analysis of drug release a challenge that requires careful consideration of the specific properties of each system [31,80].

The incorporation of antibiotics into biomaterials must not adversely affect the biocompatibility of the material. Antibiotics can sometimes elicit immune responses or cytotoxic effects that could compromise the safety and effectiveness of the biomaterial. This can occur with beta-lactams and quinolones, which causes cell necrosis and are unstable molecules to be used in additive manufacturing [100,101]. While the goal is to target localized infections, antibiotics released from biomaterials can potentially have systemic effects. Ensuring that the antibiotics act only where needed without significant systemic absorption is crucial to avoid low levels in common microbiota to avoid the selection of multidrug-resistant bacteria [102].

The side effects of antibiotics and antimicrobial metals are well-documented when used systemically, but there is a lack of studies that confirm whether their local application could lead to long-term complications after prolonged implantation. This can be a severe problem since some metals can be associated with neurotoxicity, in the case of silver nanoparticles [103]. Developing and using antibiotic-impregnated biomaterials requires extensive testing and regulatory approval. Demonstrating safety, efficacy, and consistency in the release and performance of the biomaterials is essential for regulatory acceptance.

One last challenge of biomaterials with antimicrobial activity is the high production costs. The expense of both the antibiotics and the specialized manufacturing processes can make these biomaterials more costly than their non-antibiotic counterparts.

## 9. Conclusions

In the realm of bone repair, addressing defects necessitates advanced solutions like bone grafts, crucial across medical and dental practices globally. Bovine bone scaffolds offer advantages over autogenous grafts, minimizing surgical risks and enhancing cost-effectiveness. Integrating antimicrobial properties into bone substitutes, particularly with metals like zinc, copper, and silver, holds promise for mitigating infection risks associated with graft procedures. Notably, silver nanoparticles have demonstrated robust antimicrobial efficacy against a spectrum of pathogens, while zinc nanoparticles show potential in preventing infections and supporting bone regeneration. Advances in 3D printing technology further enable the production of customized implants and scaffolds, revolutionizing treatment approaches across various medical disciplines.

Interdisciplinary collaborations continue to drive innovation in biomaterial science, offering hope for enhanced therapies addressing complex tissue damage and organ dysfunction.
